# Does mild flexion of the femoral prosthesis in total knee arthroplasty result in better early postoperative outcomes?

**DOI:** 10.1186/s12891-023-06840-w

**Published:** 2023-09-06

**Authors:** Hang Zhou, Ze-Rui Wu, Xiang-Yang Chen, Le-Shu Zhang, Jin-Cheng Zhang, Sakarie Mustafe Hidig, Shuo Feng, Zhi Yang

**Affiliations:** 1 Department of Orthopedics, The People’s Hospital of Rugao, Rugao, 226500 Jiangsu China; 2 Department of Orthopedic Surgery, Affiliated Hospital of Xuzhou Medical University, Xuzhou, 221002 Jiangsu China; 3 Department of Orthopedics, Central Laboratory, Changshu Hospital Affiliated to Soochow University, First People’s Hospital of Changshu City, Changshu, 215500 Jiangsu China

**Keywords:** Total knee arthroplasty, Knee osteoarthritis, Femoral prosthesis, Sagittal position, Femoral prosthesis flexion angle

## Abstract

**Background:**

The purpose of this study was to measure the femoral prosthesis flexion angle (FPFA) in total knee arthroplasty (TKA) using three-dimensional reconstruction, and to assess the differences in early clinical efficacy between patients with different degrees of flexion.

**Methods:**

We conducted a prospective cohort study. From June 2019 to May 2021, 113 patients admitted for TKA due to osteoarthritis of the knee were selected. The patients’ postoperative knee joints were reconstructed in three dimensions according to postoperative three-dimensional computed tomography (CT) scans. The FPFA was measured, and the patients were divided into 4 groups: anterior extension group (FPFA < 0°), mildly flexed group (0° ≤ FPFA < 3°), moderately flexed group (3° ≤ FPFA < 6°) and excessively flexed group (6° ≤ FPFA). The differences in the Knee Society Score (KSS), knee Range of Motion (ROM), and visual analogue scale (VAS) scores were measured and compared between the four groups at each postoperative time point.

**Results:**

Postoperative KSS, ROM, and VAS were significantly improved in all groups compared to the preoperative period. At 1 year postoperatively, the ROM was significantly greater in the mildly flexed group (123.46 ± 6.51°) than in the anterior extension group (116.93 ± 8.05°) and the excessively flexed group (118.76 ± 8.20°) (*P* < 0.05). The KSS was significantly higher in the mildly flexed group (162.68 ± 12.79) than in the other groups at 6 months postoperatively (*P* < 0.05). The higher KSS (174.17 ± 11.84) in the mildly flexed group was maintained until 1 year postoperatively, with a statistically significant difference (*P* < 0.05). No significant difference in VAS scores was observed between groups at each time point.

**Conclusions:**

A femoral prosthesis flexion angle of 0–3° significantly improved postoperative knee mobility, and patients could obtain better Knee Society Scores after surgery, which facilitated the postoperative recovery of knee function.

**Trial registration:**

ChiCTR2100051502, 2021/09/24.

## Background

As a treatment for end-stage osteoarthritis of the knee, TKA has achieved excellent results in relieving pain and improving knee function. With advances in materials, design concepts, and surgical techniques, TKA has become one of the most successful surgical procedures in orthopedics [1, 2]. Although most patients achieve good results after surgery, t some patients still do not achieve a satisfactory outcome after surgery. Pain relief, restoration of normal walking ability, and especially an improvement in knee mobility after TKA are particularly important in enhancing patient satisfaction and meeting the needs of daily life after surgery [3, 4].

The factors that influence postoperative ROM are both patient and surgical factors. On the patient side, influencing factors may include preoperative knee mobility, body mass index (BMI), age, sex, postoperative rehabilitation, etc. [5]. On the surgical side, it is influenced by factors such as surgical technique, type of prosthesis, soft tissue balance, patellofemoral factors, osteotomy accuracy, postoperative force line, and prosthesis angle [6, 7]. These factors can also affect other outcomes after TKA, such as knee pain [8, 9]. FPFA is one of the important sagittal parameters among surgical factors. A study by Antony et al. [10] found that FPFA was weakly positively correlated with maximum knee flexion. Scott et al. [11] noted that the flexion of the femoral prosthesis was closely related to the patient’s ability to kneel postoperatively. In addition, Kim et al. [5] stated that sagittal flexion of 0° to 3° during placement of the femoral prosthesis significantly reduced the failure rate of TKA [12]. In contrast, femoral prostheses placed in anterior extension lead to anterior knee pain after TKA [13], and predispose patients to anterior cortical notch and periprosthetic fractures [14]. Excessive flexion of the femoral prosthesis predisposes patients to aseptic loosening of the prosthesis [15] and a significantly increased risk of postoperative flexion contracture [16]. At present, most studies have focused on the effects of FPFA on long-term outcomes and complications, although the relationship between different FPFAs and patients’ early postoperative recovery is unclear.

Most of the previous studies used lateral X-rays to measure FPFA, but some errors may occur when using two-dimensional radiograph measurements due to overprinting and rotation problems. Therefore, X-ray measurement methods may be inaccurate [17]. Currently, preoperative planning and measurement of the postoperative prosthesis angle after knee arthroplasty with the help of three-dimensional CT has been shown to be more accurate than that with the help of X-rays [18–20]. Therefore, a measurement method based on three-dimensional CT scans of the postoperative knee with three-dimensional reconstruction might be more accurate for measuring FPFA. This study was based on three-dimensional reconstructions to measure FPFA after TKA. Preoperative and postoperative KSS, VAS, and ROM scores were recorded and analyzed in each group of patients during a one-year follow-up. However, as mentioned previously, many factors influence ROM after TKA as well as other outcomes. We, therefore, referred to the methodology of the study by Ruangsomboon et al. [21], appropriately controlling for all potential confounding factors, with the aim of investigating the association between different FPFAs and the early efficacy of treatment in patients undergoing TKA.

## Materials and methods

### Patient selection

This study used a prospective cohort design. Patients with osteoarthritis of the knee who were treated at the Affiliated Hospital of Xuzhou Medical University from June 2019 to May 2021 were selected, all patients underwent TKA and were operated on by the same attending surgeon. The trial has been registered with the Chinese Clinical Trials Registry (registration number: ChiCTR2100051502). The study was reviewed and approved by the local medical ethics committee (Ethics number: XYFY2021-KL312-01). Patients were informed of the need for additional postoperative knee CT scans and 3D reconstruction of the imaging data, and written informed consent was obtained.

Inclusion criteria:

1. End-stage osteoarthritis of the knee, ineffective with conservative treatment, and Kellgren-Lawtence (K-L) classification grade III-IV.
2. The patient underwent fixed-bearing posterior-stabilized (PS) TKA without patellar replacement. All patients received the same type of prosthesis from the same manufacturer and the same product.

Exclusion criteria:

1. Patients with a history of previous knee surgery or severe knee trauma.
2. Severe knee inversion (> 15°), valgus (> 10°) or flexion deformity (> 25°).
3. Patients with a history of spine, hip or ankle disease or surgery.
4. Patients who underwent revision knee surgery for joint infection during hospitalization or follow-up, or other serious complications that affected the questionnaire.
5. Incomplete follow-up data, imaging data or less than 1 year of follow-up.

### Surgery and perioperative management

All patients underwent TKA under general anesthesia. Knee pain, knee mobility, and KSS were recorded by the resident the day before surgery. The patients were operated on by the same surgeon experienced in TKA and his team to minimize the interference of differences in the surgical technique and operation on the outcome.

After anesthesia, the patient was placed in the supine position with a tourniquet tied at the root of the thigh. All surgeries were performed with a straight incision anterior to the patella and a medial approach through the parapatellar area. We used the long rod intramedullary guide on the femoral side during surgery. The entry point was generally chosen medial to the apex of the intercondylar femoral notch and above the end of the posterior cruciate ligament. Intraoperatively, the distal femur was cut at a conventional valgus of 6° using a distal osteotomy guide. The surgical trans epicondylar axis was marked during surgery. As a method to obtain a good rectangular flexion gap, the femoral prosthesis rotation is set as a slight external rotation of the prosthesis posterior condylar line relative to the surgical trans epicondylar axis, typically 3°. We performed proximal tibial cutting using the extramedullary guide. The proximal tibial osteotomy was completed using a tibial osteotomy guide with a posterior tilt angle of 5°. All patients received an appropriately sized multi-radius fixed-bearing PS femoral prosthesis (Smith & Nephew Legion, Smith and Nephew Inc., Memphis TN, USA). Preoperatively, we performed a patellar trajectory assessment based on imaging data. No patellar surface replacement was performed. Patelloplasty and peripatellar cautery were performed intraoperatively. In addition, we performed the No Thumb test during surgery. The lateral patellar support band was released if the test results indicated a possible patellar dislocation. We used a “cocktail” of an analgesic mixture for local injection and joint cavity retention (1% ropivacaine injection 200 mg, flurbiprofen ester injection 100 mg, epinephrine 1 mg and 0.9% saline).

All patients received an intravenous flurbiprofen injection and oral NSAIDs analgesia for 3 days after surgery. Dizocine was administered intramuscularly to rescue analgesia. The patients were instructed by the rehabilitation physician to walk on the floor with the assistance of a walker. Additionally, the patients were instructed to actively perform active knee flexion and extension exercises, as well as passive flexion and extension exercises of the affected limb with the help of the CPM machine. Three-dimensional CT scans (General Electric Optima 660 64-row model, USA) of the knee in the supine position were taken within 1 week after TKA in all patients. The scanned data were saved in DICOM format. At the time of discharge, we explained to the patients the relevant precautions and instructed them to perform post-discharge rehabilitation training according to the instructions provided during their stay in the hospital.

### Outcome indicators

#### Prosthesis angles

The three-dimensional CT imaging data were imported into Mimics 21 software as DICOM files. The Edit Masks function was used to fill in the areas that were not automatically covered by the mask and remove the excess areas. The processed masks were exported as three-dimensional models. The model was imported into Geomagic software and further processed using functions such as packing and smoothing to obtain a smooth geometric model of the knee joint. Measurements were performed using Solid Works 2018 software.

**Fig. 1:**
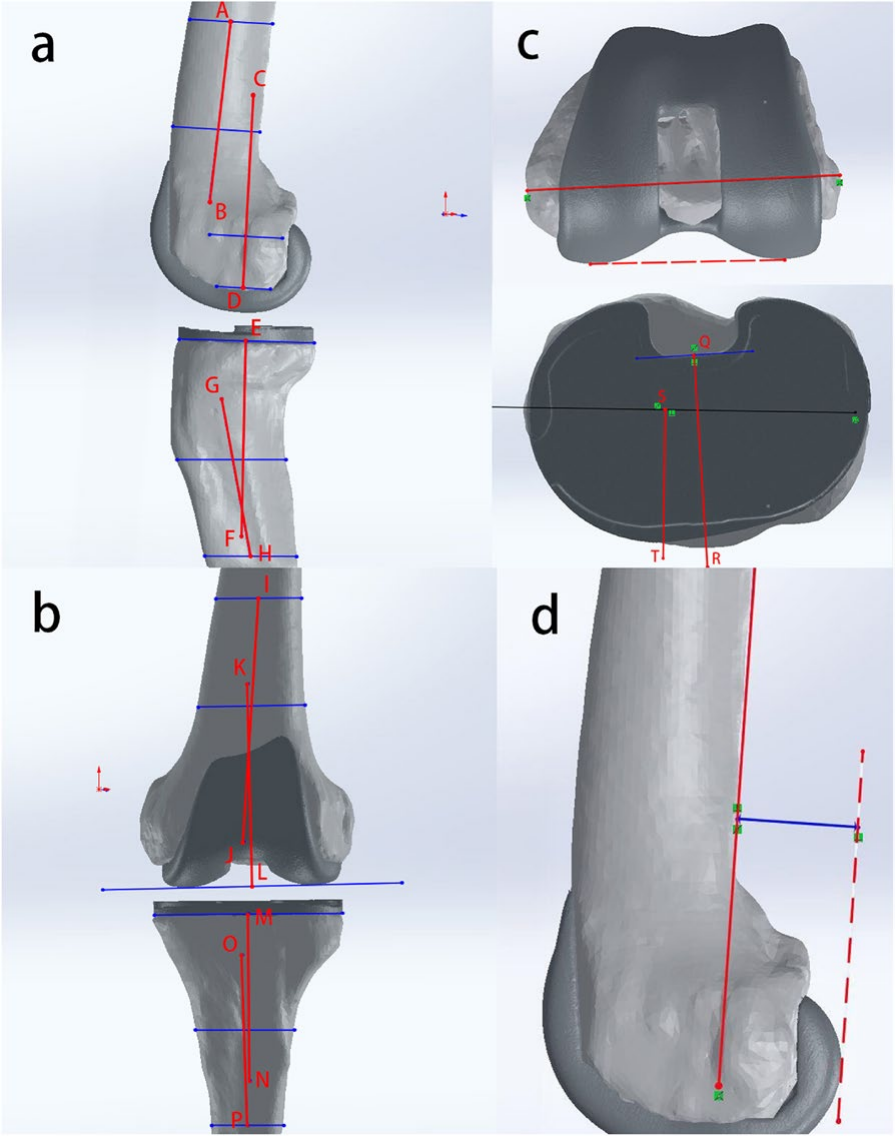
Measurement of prosthesis angles, TFA and PCO. **a** shows the sagittal plane of the knee joint, AB is the anatomical axis of the distal femur, and CD is the longitudinal axis of the femoral prosthesis. The angle between the two is the FPFA. The angle between EF and GH is the tibial prosthesis slope. **b** shows the coronal plane of the knee. The angle between IJ and KL is the femoral prosthesis valgus angle. The angle between MN and OP is the tibial prosthesis valgus angle. The angle between IJ and OP is the TFA. **c** the red solid line is the surgical transepicondylar axis, and the dotted line is the posterior condylar line of the femoral prosthesis. The angle between the two is the femoral prosthesis rotation angle. The angle between the vertical line (ST) of the surgical transepicondylar axis and QR is the tibial prosthesis rotation angle. **d** the distance between the tangent line of the posterior femoral cortex (red solid line) and the tangent line of the posterior condyle apex (red dashed line) is the PCO

The FPFA was measured along with the tibial prosthesis slope, the tibial and femoral prosthesis valgus angle, the rotation of the tibial and femoral prostheses, and the posterior condylar offset (PCO) to exclude the effects of the remaining angles of the prosthesis on the outcome. Our measurements were performed with reference to previous literature using X-ray radiographs to measure each angle of the postoperative prosthesis after TKA [20, 22–24]. The three-dimensional model was adjusted to true lateral, true coronal, and true cross-sectional positions. (1) The angle between the anatomical axis of the distal femur and the longitudinal axis of the femoral prosthesis in the lateral plane is the FPFA, with flexion indicated as positive and extension as negative when recording the values. The angle between the anatomical axis of the proximal tibia and the longitudinal axis of the tibial prosthesis is the tibial prosthesis slope, with the posterior slope being positive and the anterior slope being negative. (2) The angle between the distal anatomic axis of the femur and the vertical line of the distal tangent of the femoral prosthesis in the coronal position is the femoral prosthesis valgus angle, and the angle between the proximal anatomic axis of the tibia and the longitudinal axis of the tibial prosthesis is the tibial prosthesis valgus angle. The angle between the anatomical axis of the tibia and the anatomical axis of the femur is the anatomical tibiofemoral axis (TFA), with the valgus recorded as positive and the varus as negative. (3) The angle between the surgical trans epicondylar axis and the posterior condylar line of the femoral prosthesis in cross-section is the femoral prosthesis rotation angle, and the angle between the vertical line of the surgical trans epicondylar axis and the anterior-posterior axis of the tibial prosthesis is the tibial prosthesis rotation angle, with external rotation recorded as positive and internal rotation as negative. (4) The distance between the tangent line of the posterior femoral cortex and the tangent line of the posterior condyle apex is the PCO (Fig. 1). According to the postoperative FPFA, the patients were divided into 4 groups: anterior extension group (FPFA < 0°), mildly flexed group (0° ≤ FPFA < 3°), moderately flexed group (3° ≤ FPFA < 6°) and excessively flexed group (6° ≤ FPFA).

#### Knee range of motion (ROM)

The patient is placed prone on a rigid bed and the knee is flexed and extended to its maximum. The angle between the longitudinal axis of the femur and the longitudinal axis of the tibia is measured in the sagittal plane, which is the knee ROM.

#### Knee Society Score (KSS)

The KSS consists of a clinical score and a functional score; the clinical score totals 100 points, including pain (50 points), mobility (25 points), stability (25 points) and subtractive items (flexion contracture, extension lag, and alignment). The functional score is 100 points and includes the ability to walk (50 points), the ability to walk up and down stairs (50 points) and subtractive items (the need for assistance when walking). A higher score indicates less knee pain and better function.

#### Visual analogue scale (VAS) score

A Vernier scale with 10 scales of approximately 10 cm in length is used, with scales of “0” and “10” noted at each end. A score of 0 indicates no pain, and 10 indicates the most severe pain that is unbearable. The patient faces the unscaled side of the scale, and the Vernier scale is placed on the area that best represents the pain level at that time; the doctor assigns the patient a score based on the location marked.

All measurements and assessments were performed by two residents of the same seniority. Preoperative age, sex, right or left side, and BMI were recorded for all four groups. ROM, KSS, and VAS scores were measured and recorded on the day before surgery in all four groups. After routine TKA, patients received follow-up with outpatient reviews and phone calls at 2 weeks, 1 month, 3 months, 6 months, and 12 months postoperatively to record ROM, KSS, and VAS scores of the knee.

Based on a study by Antony et al. [10] assessing the effect of sagittal alignment of the TKA prosthesis on knee kinematics, we considered that a minimum sample size of 105 cases would be required to generate 80% statistical power to test for a moderate level of the effect size when α was taken as 0.05 and tested bilaterally. We planned to increase the number of cases by an additional 15% and expected to include a minimum of 120 knees to avoid missed follow-ups and cases excluded for any reason.

### Statistical analysis

The data obtained in this study were analyzed using SPSS Statistics 25.0 statistical software (SPSS Inc., Chicago, IL). The data measured by two physicians separately were tested for consistency and evaluated using the intraclass correlation coefficient (ICC), with an ICC > 0.80 judged as a high consistency of measurement. The Shapiro‒Wilk test was used to test for normality of age, BMI, KSS, ROM, VAS score, each angle of the knee prosthesis, TFA, and PCO.

The comparisons of KSS, ROM, and VAS scores at different time points within each group were analyzed using repeated-measures ANOVA. An independent sample nonparametric test was used for each measure that was not normally distributed between groups. One-way ANOVA was used for each measure that met the normal distribution. Post hoc comparisons were performed using the least significant difference (LSD) method. Count data such as sex and right or left side were analyzed using the chi-square test or Fisher’s exact probability method. The test level α value was set to 0.05 for both sides, and the difference was considered statistically significant at *P* < 0.05. In addition, we performed a post hoc power analysis to determine that more than 80% power was needed to detect statistically significant differences at the 0.05 level.

## Results

### Baseline information and prosthesis angle

**Fig. 2:**
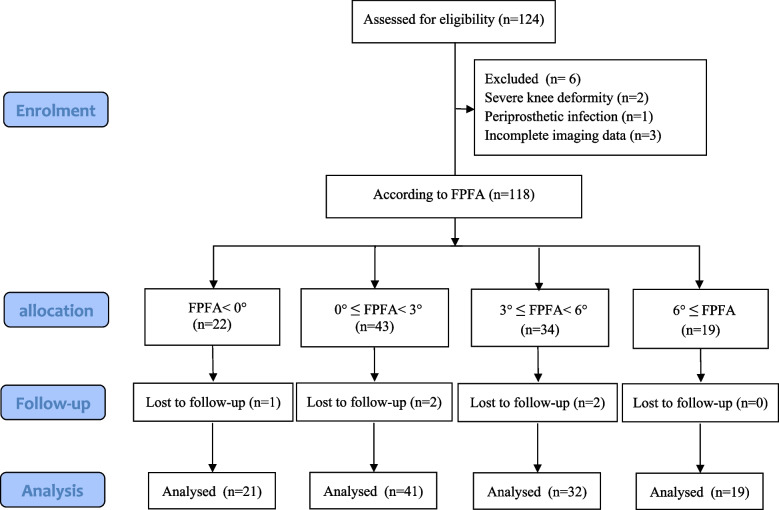
Flow diagram of patient enrolment and assignment

The ICCs of all measurements recorded by the two physicians were > 0.8. The data measured by the two physicians were in good agreement and the results were highly reliable. A total of 124 patients met the inclusion criteria. 2 patients with a severe knee deformity, 1 patient with postoperative periprosthetic infection requiring revision surgery, 3 patients with incomplete imaging data, and 5 patients who were lost to follow-up were excluded. Cases lost to follow-up were patients who could not be contacted, and those who did not come to the hospital for a review within the required time frame after notification of the review or did not finish a complete efficacy assessment. These cases were eventually excluded. No informative missing cases occurred in the study. A total of 113 patients were included in the study after screening and exclusion (Fig. 2). The FPFA of patients after TKA was measured, and the patients were divided into 4 groups. 21 patients with FPFA < 0° were assigned to the anterior extension group. 41 patients with 0° ≤ FPFA < 3° were assigned to the mildly flexed group. 32 patients with 3 ≤ FPFA < 6° were assigned to the moderately flexed group, and 19 patients with 6° ≤ FPFA were assigned to the excessively flexed group.

No significant differences in age, BMI, sex, and right or left sides of surgery were observed between the four groups (all *P* > 0.05) (Table 1). The mean values for the FPFA in the anterior extension, neutral position, mildly flexed, and excessively flexed groups were − 1.54°, 1.87°, 4.40°, and 7.30°, respectively. Significant differences were not observed between the four groups in the femoral and tibial prosthesis valgus angle, tibial prosthesis slope, and femoral and tibial prosthesis rotation angle (all *P* > 0.05). The postoperative PCO was significantly greater in the excessively flexed group than in the other three groups, and the postoperative PCO was greater in the mildly flexed and moderately flexed groups than in the anterior extension group (*P* < 0.05) (Table 2). No significant differences in preoperative KSS, ROM, and VAS scores were observed between groups (*p* > 0.05).

**Table 1:** Comparison of baseline information

	Anterior extension group	Mildly flexed group	Moderately flexed group	Excessively flexed group	Test value	*P* value
Number	21	41	32	19	-	-
Age($\overline{\mathrm x}$ ± s, years)	64.67 ± 8.71	65.24 ± 6.77	66.38 ± 7.58	64.21 ± 7.77	F = 0.398	0.755
BMI($\overline{\mathrm x}$ ± s,kg/m^2^)	26.87 ± 3.29	27.51 ± 2.63	28.20 ± 2.61	27.68 ± 3.95	F = 0.852	0.468
Side(R/L)	13/8	21/20	15/17	7/12	χ^2^ = 2.647	0.449
Gender (M/F)	6/15	13/28	9/23	8/11	χ^2^ = 1.229	0.746

**Table 2:** Comparison of knee prosthesis angle, TFA and PCO between groups ($\overline{\mathrm x}$ ± s)

	Anterior extension group	Mildly flexed group	Moderately flexed group	Excessively flexed group	F/H	P value
Number	21	41	32	19	-	-
femoral prosthesis valgus angle(°)	5.31 ± 1.84	4.90 ± 1.76	5.12 ± 1.71	5.66 ± 1.29	0.939	0.425
femoral prosthesis rotation angle(°)	3.54 ± 0.97	3.48 ± 1.28	3.52 ± 1.26	3.06 ± 1.48	0.669	0.573
tibial prosthesis slope(°)	5.97 ± 1.33	6.34 ± 1.54	5.98 ± 1.48	6.18 ± 1.54	0.459	0.712
tibial prosthesis valgus angle(°)	-0.28 ± 2.07	0.16 ± 2.44	0.18 ± 2.21	-0.24 ± 2.13	0.329	0.804
tibial prosthesis rotation angle(°)	3.05(1.09, 3.87)	3.22(1.52, 4.08)	3.95(2.11, 4.70)	2.91(1.23, 3.47)	3.555	0.314
TFA(°)	5.45 ± 2.97	4.53 ± 3.38	4.66 ± 2.37	4.88 ± 3.17	0.455	0.714
PCO(mm)	28.85 ± 3.19	30.80 ± 2.08^*^	31.62 ± 2.97^a^	33.66 ± 2.42^b^	11.678	< 0.001

^*^, ^a^,^b^ indicate statistically significant differences (*P* < 0.05)

^*^P = 0.007 compared to the anterior extension group

^a^*P* < 0.001 compared to the anterior extension group

^b^*P* < 0.001 compared to the anterior extension group, *P* < 0.001 compared to the mildly flexed group, and *P* = 0.008 compared to the moderately flexed group

### ROM

**Fig. 3:**
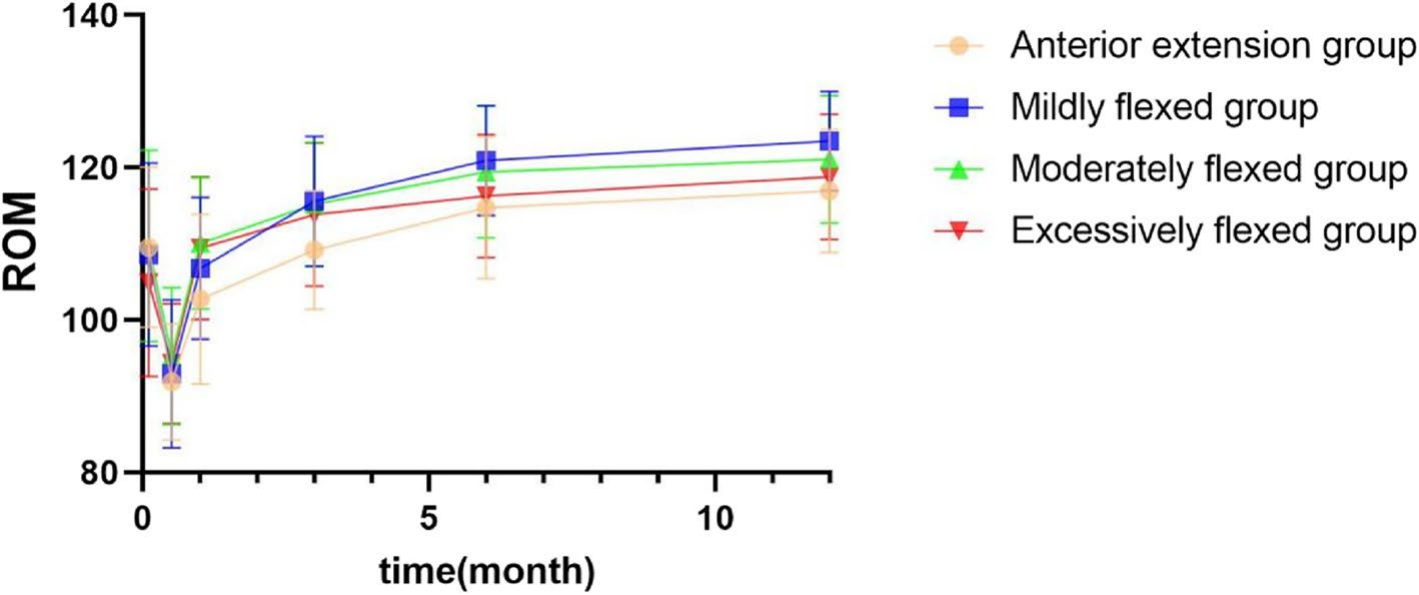
Comparison of ROM before and after surgery and between groups

Postoperative activity was significantly higher in all groups compared to preoperative levels (*P* < 0.05). Transient decreases were noted at 2 weeks postoperatively, and all patients recovered to preoperative levels after 3 months postoperatively. At 1 month postoperatively, ROM was significantly lower in the anterior extension group than in the moderately flexed group (*p* = 0.007) and excessively flexed group (*p* = 0.029). At 3 months postoperatively, a greater ROM began to be observed in the mildly flexed group than in the anterior extension group (*P* = 0.005), while a significant difference was no longer observed between the excessively flexed group and the anterior extension group. At the final follow-up at 1 year postoperatively, significantly greater ROM was recorded for patients in the mildly flexed group than in the anterior extension group (*P*=0.002) and the excessively flexed group (*P*=0.029) (Table 3; Fig. 3).

**Table 3:** Comparison of ROM before and after surgery and between groups ($\overline{\mathrm x}$ ± s, °)

	Anterior extension group	Mildly flexed group	Moderately flexed group	Excessively flexed group	F	P value
Number	21	41	32	19	-	-
Pre-op	109.53 ± 10.48	108.56 ± 12.03	109.70 ± 12.59	104.89 ± 12.31	0.732	0.535
2 weeks post-op	91.86 ± 7.63	92.92 ± 9.72	95.26 ± 8.96	94.26 ± 7.86	0.762	0.518
1 month post-op	102.72 ± 11.17	106.79 ± 9.34	110.09 ± 8.68^*^	109.40 ± 9.37^a^	2.873	0.040
3 months post-op	109.15 ± 7.83	115.58 ± 8.51^*^	115.17 ± 8.08^a^	113.83 ± 9.42	3.006	0.033
6 months post-op	114.70 ± 9.30	120.92 ± 7.20^*^	119.44 ± 8.68^a^	116.28 ± 8.08	3.303	0.023
1 year post-op	116.93 ± 8.05	123.46 ± 6.51^*^	121.07 ± 8.36	118.76 ± 8.20	3.919	0.011
F	42.895	119.445	58.449	41.705	-	-
*P* value	0.000	0.000	0.000	0.000	-	-

*ROM* Knee range of motion

^*^, ^a^ indicates statistically significant differences between two groups at the same time point (*P* < 0.05)

At 1 month postoperatively, ^*^P = 0.007 compared to the anterior extension group and ^a^*P* = 0.029 compared to the anterior extension group

At 3 months postoperatively, ^*^P = 0.005 compared to the anterior extension group and ^a^*P* = 0.012 compared to the anterior extension group

At 6 months postoperatively, ^*^P = 0.006 compared to the anterior extension group, *P* = 0.044 compared to the excessively flexed group, and ^a^*P* = 0.042 compared to the anterior extension group

At 1 year postoperatively, ^*^P = 0.002 compared to the anterior extension group, and *P* = 0.029 compared to the excessively flexed group

### Comparison of KSS among groups

#### KSS clinical score

**Fig. 4:**
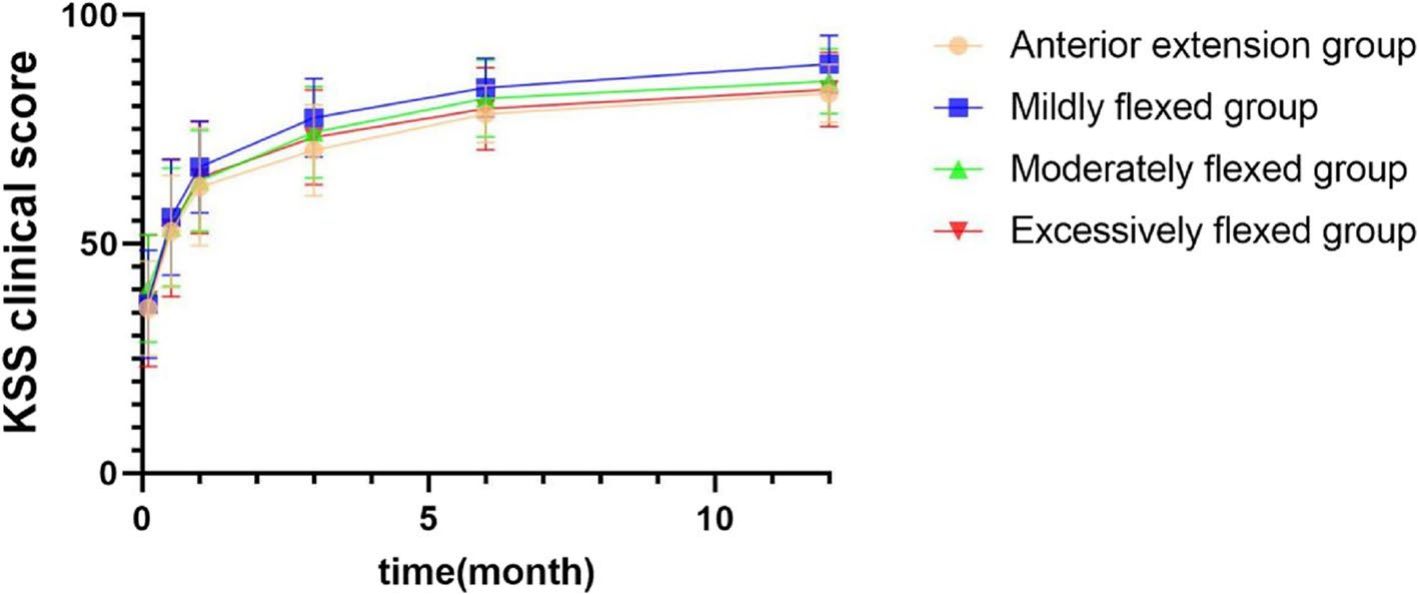
Comparison of KSS clinical score before and after surgery and between groups

The postoperative KSS clinical scores were significantly higher in all groups compared to the preoperative period (*P* < 0.05). The KSS clinical scores were significantly higher in the mildly flexed group than in the anterior extension group at 3 months postoperatively (*P* = 0.006). The KSS clinical scores were significantly higher in the mildly flexed group than in the anterior extension group (*P* = 0.005) and excessively flexed group (*P* = 0.029) at 6 months postoperatively. At 1 year postoperatively, the KSS clinical scores were significantly higher in the mildly flexed group than in the anterior extension group (*P* = 0.001), moderately flexed group (*P* = 0.021), and excessively flexed group (*P* = 0.004) (Table 4; Fig. 4).

**Table 4:** Comparison of KSS clinical score before and after surgery and between groups ($\overline{\mathrm x}$ ± s)

	Anterior extension group	Mildly flexed group	Moderately flexed group	Excessively flexed group	F	P value
Number	21	41	32	19	-	-
Pre-op	35.95 ± 10.23	36.85 ± 11.78	40.25 ± 11.67	37.63 ± 14.37	0.702	0.553
2 weeks post-op	52.71 ± 12.24	55.83 ± 12.62	53.59 ± 12.99	53.47 ± 15.01	0.345	0.793
1 month post-op	62.43 ± 12.89	66.78 ± 10.03	63.78 ± 10.99	64.42 ± 12.19	0.829	0.480
3 months post-op	70.43 ± 9.94	77.56 ± 8.59^*^	74.38 ± 10.03	73.26 ± 10.34	2.770	0.045
6 months post-op	78.38 ± 6.29	84.15 ± 6.43^*^	81.78 ± 8.45	79.53 ± 9.03	3.355	0.022
1 year post-op	82.86 ± 6.33	89.29 ± 6.29^*^	85.50 ± 7.15	83.74 ± 8.08	5.342	0.002
F	145.395	277.675	184.637	98.262	-	-
*P* value	0.000	0.000	0.000	0.000	-	-

*KSS* Knee society score

^*^ indicates statistically significant differences between two groups at the same time point (*P* < 0.05)

At 3 months postoperatively, ^*^P = 0.006 compared to the anterior extension group

At 6 months postoperatively, ^*^P = 0.005 compared to the anterior extension group and *P* = 0.029 compared to the excessive flexed group

At 1 year postoperatively, ^*^P = 0.001 compared to the anterior extension group, *P* = 0.021 compared to moderately flexed group, and *P* = 0.004 compared to excessively flexed group

#### KSS functional score

**Fig. 5:**
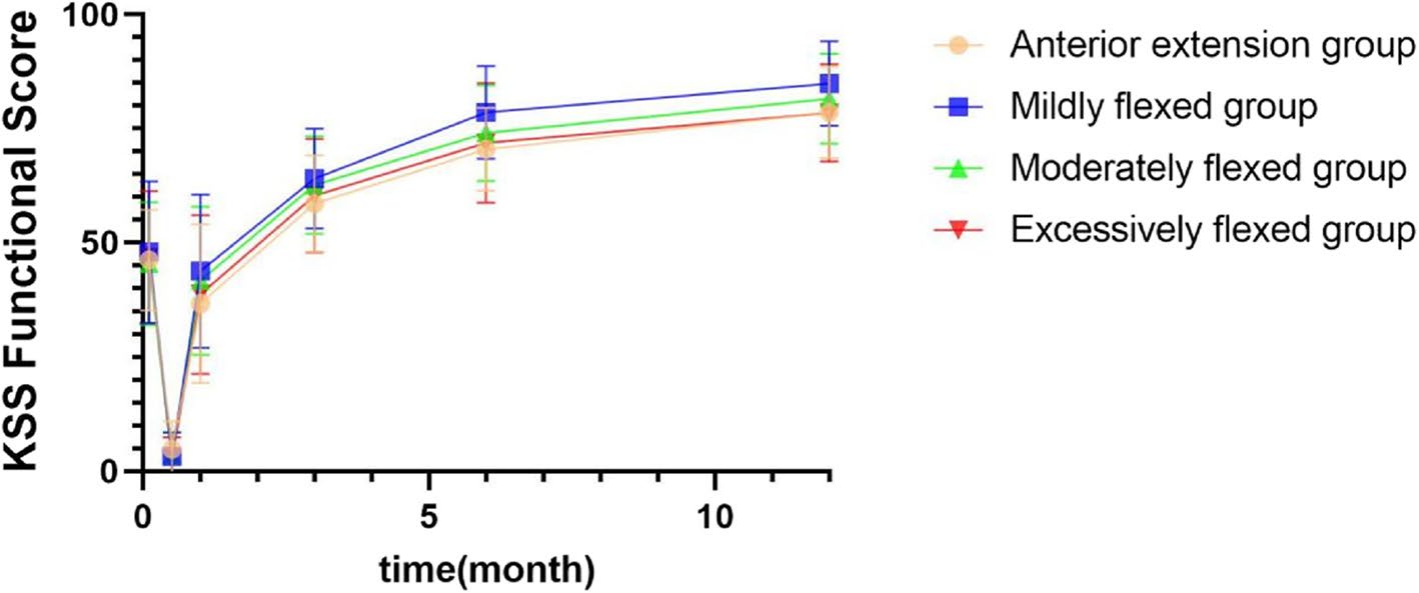
Comparison of KSS functional score before and after surgery and between groups

The KSS functional scores were significantly higher in all four groups postoperatively than preoperatively (all *P* < 0.05). The KSS functional scores decreased significantly at 2 weeks postoperatively and recovered to values greater than the preoperative levels at 3 months postoperatively. At 6 months postoperatively, KSS functional scores were significantly higher in the mildly flexed group than in the anterior extension group (*P* = 0.006) and excessively flexed group (*P* = 0.025). At 1 year postoperatively, the KSS functional score was higher in the mildly flexed group than in the anterior extension group (*P* = 0.019) and excessively flexed group (*P* = 0.020) (Table 5; Fig. 5).

**Table 5:** Comparison of KSS functional score before and after surgery and between groups ($\overline{\mathrm x}$ ± s)

	Anterior extension group	Mildly flexed group	Moderately flexed group	Excessively flexed group	F	*P* value
Number	21	41	32	19	-	-
Pre-op	46.19 ± 11.05	47.93 ± 15.53	45.47 ± 13.46	46.84 ± 14.45	0.196	0.899
2 weeks post-op	4.76 ± 6.22	3.17 ± 5.33	3.59 ± 4.96	2.63 ± 4.82	0.617	0.606
1 month post-op	36.67 ± 17.34	43.78 ± 16.80	41.72 ± 16.19	38.68 ± 17.39	0.976	0.407
3 months post-op	58.57 ± 10.62	64.02 ± 10.97	62.66 ± 10.70	60.26 ± 12.41	1.319	0.272
6 months post-op	70.48 ± 9.07	78.54 ± 10.14^*^	74.06 ± 10.51	71.84 ± 13.15	3.388	0.021
1 year post-op	78.57 ± 10.14	84.88 ± 9.25^*^	81.56 ± 9.87	78.42 ± 10.68	2.838	0.041
F	199.364	360.597	282.594	114.395	-	-
*P* value	0.000	0.000	0.000	0.000	-	-

*KSS* Knee society score

^*^, ^a^ indicates statistically significant differences between two groups at the same time point (*P* < 0.05)

At 6 months postoperatively, ^*^P = 0.006 compared to the anterior extension group and *P* = 0.025 compared to the excessively flexed group

At 1 year postoperatively, ^*^P = 0.019 compared to the anterior extension group and *P* = 0.020 compared to the excessively flexed group

### KSS

**Fig. 6:**
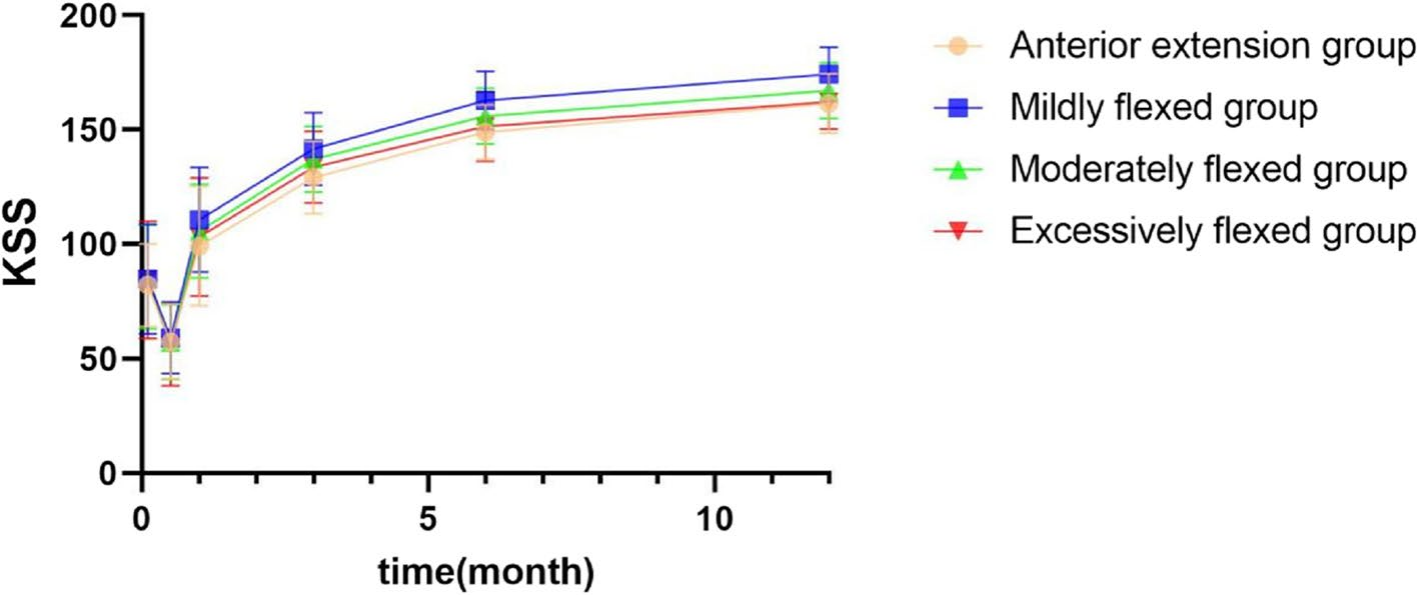
Comparison of KSS before and after surgery and between groups

The differences between the KSS of each group before and after surgery were statistically significant (all *p* < 0.05). The KSS decreased at 2 weeks postoperatively compared with the preoperative period and then gradually increased, mainly due to the decrease in KSS functional scores. The KSS was significantly higher in the mildly flexed group than in the anterior extension group (*P* = 0.003) at 3 months postoperatively. At 6 months postoperatively, the KSS was significantly higher in the mildly flexed group than in the anterior extension group (*P* < 0.001), the moderately flexed group (*P* = 0.027), and the excessively flexed group (*P* = 0.002). The KSS was significantly higher in the mildly flexed group than in the anterior extension group (*P* < 0.001), the moderately flexed group (*P* = 0.015), and the excessively flexed group (*P* = 0.001) at 1 year postoperatively (Table 6; Fig. 6).

**Table 6:** Comparison of KSS before and after surgery and between groups ($\overline{\mathrm x}$ ± s)

	Anterior extension group	Mildly flexed group	Moderately flexed group	Excessively flexed group	F	*P* value
Number	21	41	32	19	-	-
Pre-op	82.14 ± 18.14	84.78 ± 23.90	85.72 ± 22.69	84.47 ± 25.59	0.106	0.956
2 weeks post-op	57.48 ± 16.42	59.00 ± 15.60	57.19 ± 16.27	56.11 ± 17.84	0.159	0.924
1 month post-op	99.10 ± 25.99	110.56 ± 22.85	105.50 ± 20.56	103.11 ± 25.82	1.230	0.302
3 months post-op	129.00 ± 15.89	141.59 ± 15.91^*^	137.03 ± 14.41	133.53 ± 15.74	3.370	0.021
6 months post-op	148.86 ± 12.02	162.68 ± 12.79^*^	155.84 ± 12.29	151.37 ± 15.28	6.550	< 0.001
1 year post-op	161.43 ± 13.06	174.17 ± 11.84^*^	167.06 ± 12.15	162.16 ± 11.91	7.061	< 0.001
F	248.481	458.780	351.189	147.725	-	-
*P* value	0.000	0.000	0.000	0.000	-	-

*KSS* Knee society score

^*^, ^a^,^b^ indicates statistically significant differences between two groups at the same time point (*P* < 0.05)

At 3 months postoperatively, ^*^P = 0.003 compared to the anterior extension group

At 6 months postoperatively, ^*^P < 0.001 compared to the anterior extension group, *P* = 0.027 compared to the moderately flexed group, and *P* = 0.002 compared to the excessively flexed group

At 1 year postoperatively, ^*^P < 0.001 compared to the anterior extension group, *P* = 0.015compared to the moderately flexed group, and *P* = 0.001 compared to the excessively flexed group

### VAS scores during activity

**Fig. 7:**
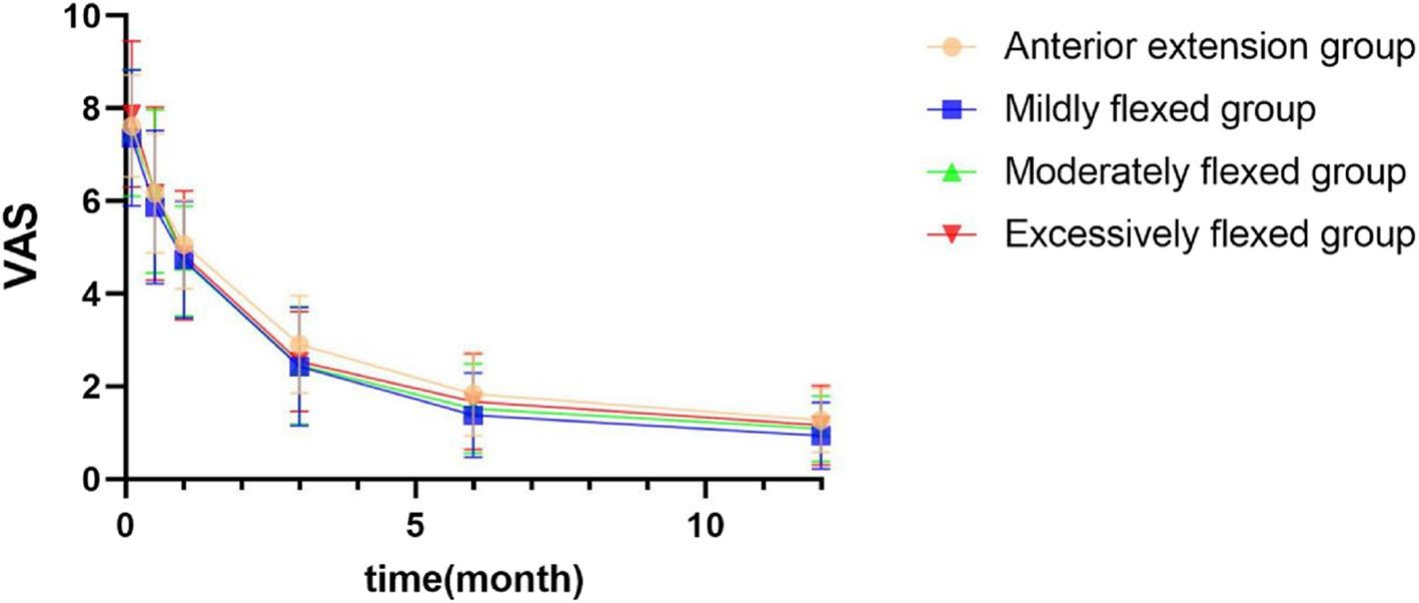
Comparison of VAS before and after surgery and between groups

The differences in VAS scores during activity before and after surgery were statistically significant in all groups (*p* < 0.05), and the VAS score was significantly lower in all groups at 1 year postoperatively compared to preoperatively. No statistically significant difference in VAS scores was observed between groups at all postoperative time points (*p* > 0.05) (Table 7; Fig. 7).

**Table 7:** Comparison of VAS before and after surgery and between groups ($\overline{\mathrm x}$ ± s)

	Anterior extension group	Mildly flexed group	Moderately flexed group	Excessively flexed group	F	*P* value
Number	21	41	32	19	-	-
Pre-op	7.62 ± 1.10	7.36 ± 1.47	7.47 ± 1.37	7.88 ± 1.58	0.640	0.591
2 weeks post-op	6.17 ± 1.28	5.87 ± 1.65	6.21 ± 1.76	6.16 ± 1.87	0.322	0.810
1 month post-op	5.06 ± 0.96	4.74 ± 1.26	4.70 ± 1.19	4.83 ± 1.39	0.433	0.730
3 months post-op	2.90 ± 1.05	2.43 ± 1.28	2.45 ± 1.27	2.54 ± 1.08	0.799	0.497
6 months post-op	1.84 ± 0.90	1.38 ± 0.91	1.52 ± 0.97	1.67 ± 1.03	1.224	0.305
1 year post-op	1.27 ± 0.69	0.94 ± 0.72	1.09 ± 0.71	1.16 ± 0.86	1.040	0.378
F	153.103	220.366	175.792	93.016	-	-
*P* value	0.000	0.000	0.000	0.000	-	-

*VAS* Visual analog score

## Discussions

The most important finding of this study was that different levels of flexion of the femoral prosthesis after TKA in patients correlated with postoperative ROM and KSS in patients, but a significant association with the VAS score was not observed.

Compared to X-ray radiographs, three-dimensional CT scans are significantly superior in preoperative planning and postoperative evaluations of TKA [18–20]. Ueyama et al. [19] reported poor agreement between two-dimensional and three-dimensional measurements in identifying abnormal values. A two-dimensional evaluation may mask or underestimate postoperative prosthetic misalignment. A three-dimensional evaluation after TKA is necessary to accurately assess the postoperative component position. Therefore, we performed a three-dimensional reconstruction of the patient’s knee postoperatively for more accurate measurements and grouping. The effects of each angle of the knee prosthesis on the experimental results were also excluded. In the present study, statistically significant differences in the femoral and tibial prosthesis valgus angle, tibial prosthesis slope, femoral and tibial prosthesis rotation angle, and TFA were not observed between the four groups compared (*P* < 0.05).

Scott et al. [13] reported that femoral prosthesis extension was one of the independent predictors of anterior knee pain 10 years after TKA. Kohet al. [25] noted that as femoral prosthesis flexion increased, posterior tibiofemoral excursion increased, and quadriceps muscle strength and knee joint pressure gradually decreased. When the femoral prosthesis is excessively flexed, the anterior end of the prosthesis cuts into the patella, resulting in a microfracture of the patella and thus anterior knee pain [26]. Theoretically, both anterior extension and excessive flexion of the femoral prosthesis can lead to patellar or prosthetic wear, causing patellar tendon damage and resulting in anterior knee pain after TKA. However, the one-year follow-up of our study did not reveal any difference in postoperative pain scores between the groups. The explanation for this result may be that the follow-up period was too short, and a longer follow-up should be considered to assess the pain scores. ROM and KSSs in all groups showed a decrease at 2 weeks postoperatively, mainly due to the patient’s postoperative knee pain and being in the early recovery period. The early KSSs were also affected by the reduction in scores caused by using single and double crutches and walking frames after surgery. The anterior extension group, moderately flexed group, and excessively flexed group had lower KSS clinical scores than the mildly flexed groups due to pain, decreased range of motion, and hyperextension or flexion contractures. These same factors also affected their KSS functional scores, resulting in poor overall KSSs. This result is similar to some previous studies. Nishitani et al. [24] found that patients’ 2011 KSSs, satisfaction, expectations, and functional activity were significantly reduced when the femoral prosthesis was placed in excessive flexion. Previously conducted mechanical studies on the sagittal angle of the femoral prosthesis noted that knee joint stresses decreased with increasing femoral prosthesis flexion [25]. A study by Antony et al. [10] found that FPFA was weakly positively correlated with maximum knee flexion. Murphy et al. [27] found that flexion femoral prosthesis placement immediately increased knee flexion and increased posterior condylar displacement. Proper flexion of the femoral prosthesis reduces the amount of osteotomy of the posterior femoral condyle, which in turn avoids a reduction in the PCO. The recovery of PCO delays impingement between the posterior femoral cortex and the posterior edge of the tibial plateau, which theoretically results in better postoperative knee mobility. Flexion of the femoral prosthesis also avoids overfilling of the patellofemoral joint, tension of the knee extension component, or irritation of the surrounding soft tissues by the lateral protrusion of the prosthesis due to the choice of a larger prosthesis for PCO reconstruction [28, 29]. This finding is generally consistent with the results of our study. We observed a significantly greater postoperative PCO in the excessively flexed group than in the other three groups, and the postoperative PCO was significantly greater in the mildly flexed and moderately flexed groups than in the anterior extension group. In the early postoperative period, postoperative ROM was higher in the moderately flexed and excessively flexed groups. However, after 3 months postoperatively, the ROM in the mildly flexed group began to show a predominance that persisted until 1 year postoperatively. In contrast, ROM was more limited in the moderately flexed and excessively flexed groups at 1 year postoperatively, especially in the excessively flexed group. We considered that this outcome was mainly due to reduced extension function in some patients in the moderately flexed and excessively flexed groups who had excessive femoral prosthesis flexion angles. At the 1-year postoperative follow-up, eleven patients in the moderately flexed and excessively flexed groups still had persistent knee extension limitations of more than 5°. According to Lustig et al. [16], patients with sagittal flexion of the femoral prosthesis > 3.5° from the mechanical axis had a 2.9-fold increased risk of flexion contracture at the one-year follow-up. In patients with knee flexion contractures, a significant amount of energy is required from the quadriceps to help load and stabilize the knee. This large amount of energy may lead to abnormal fatigue from standing, walking, and climbing stairs, reducing overall knee function [30]. The previous literature also indicates that flexion of the femoral prosthesis leads to an increased risk of arthroplasty failure and decreased outcomes. In a study by Kim et al. [12], the TKA failure rate was significantly higher in patients with a femoral prosthesis flexion > 3°. Excessive sagittal flexion of the femoral prosthesis may lead to overfilling of the patellofemoral joint during TKA or anterior impingement between the tibial prosthesis column and the femoral prosthesis intercondylar box, resulting in polyethylene liner wear and cam-post engagement [31]. During the follow-up period of this study, no cases of aseptic loosening of the prosthesis after knee arthroplasty that required revision surgery were documented. Additional follow-up studies are needed to examine patients’ postoperative periprosthetic femoral fractures, prosthetic loosening, and wear.

Many factors contribute to the different sagittal flexion angles of the femoral prosthesis. Chung et al. [32] measured the mechanical axis and the anatomical axis of the distal femur in 200 lateral femoral films and found that the difference between the two axes increased with increasing distal femoral flexion and increasing femoral head length. The distal femoral osteotomy angle assisted by computerized navigation techniques was generally perpendicular to the mechanical axis in the sagittal plane of the femur. Because of the anterior bowing angle of the femur, osteotomies performed in this manner, as well as placed femoral prostheses, often equate to the anatomic axis of the distal femur being in anterior extension [33]. The opening position and deviation [34] of the intramedullary femoral alignment bar affect the coronal and sagittal alignment of the femoral prosthesis. Short femurs generally have a large anterior femoral bowing angle, and when a long femoral medullary positioning rod is placed, the rod will automatically move forwards to correct the anterior femoral bowing angle, thus predisposing the femoral prosthesis to postoperative placement in an anterior extension position [35, 36]. In these patients, flexion of the femoral prosthesis implant may avoid the occurrence of anterior femoral cortical notching [33]. In addition, the operator’s surgical proficiency and the amount of osteotomy of the posterior femoral condyle also potentially cause the femoral prosthesis to be placed in flexion or extension [37]. After considering these factors, Kuriyama et al. [38] suggested that the sagittal position of the femoral component should be aimed at the anatomical rather than the mechanical axis. The patients’ height should be measured, and full-length films of the lower extremity in the standing position should be captured routinely to assess the anterior femoral bowing angle and femoral length before surgery and to obtain the appropriate sagittal position of the femoral prosthesis. For patients with a short height, short femur, and large anterior femoral bowing angle, we should avoid inserting the intramedullary positioning rod too deeply or using short intramedullary rods for positioning. Adjustment of the distal femoral osteotomy angle is also required when using navigation-assisted techniques in these patients. For those with a small anterior femoral bowing angle and a long femur, slightly forwarding the opening of the intramedullary positioning rod may be employed. Intraoperatively, the amount of posterior condylar osteotomy should be controlled, and the posterior condylar osteophyte should be carefully removed, which are also factors affecting FPFA. In addition, robot-assisted techniques have been used in recent years to assist in TKA. Robot-assisted TKA allows for more precise osteotomies, better postoperative prosthesis position, better force line recovery, and prolonged prosthesis life [39].

The results of our study showed that patients recovered better postoperative ROM and KSS when the femoral prosthesis was placed in 0–3° of flexion. Although hyperextension placement of the prosthesis causes limited knee flexion, hyperflexion of the femoral prosthesis causes limited knee extension. Both prosthesis angles result in a decrease in overall knee mobility and do not facilitate the recovery of postoperative KSSs. This study may provide a clinical reference for the sagittal angle during intraoperative femoral prosthesis implantation. We recommend placing the femoral prosthesis in a mildly flexed sagittal position of 0–3° to provide better postoperative knee mobility and function.

### Limitations

Our study has some limitations. First, the sample size of this study was small, especially the number of patients in the anterior extension group and excessively flexed group. Studies with larger sample sizes are needed. Many factors are thought to influence knee motion and function after TKA. We carefully designed this prospective study to minimize the effects of confounding factors. Nonetheless, we still cannot guarantee that we completely controlled for the effects of all confounding factors on the experimental results. Future studies should strive to control confounding factors to a greater extent. Due to the different sagittal morphologies of the different prostheses, only one type of prosthesis was studied in this study to reduce the effects of prosthetic factors on the results. Therefore, our findings may not be generalized to all other prostheses. In addition, the follow-up period for this study was only one year, which is relatively short. Longer follow-up is needed to assess the effects of long-term complications such as flexion contracture, implant loosening and incision-related fractures, as well as pain.

## Conclusions

A femoral prosthesis flexion angle of 0–3° significantly improved postoperative knee mobility, and patients obtained better Knee Society scores after surgery, which facilitated postoperative recovery of knee function.

## Data Availability

The data that support the findings of this study are available from Affiliated Hospital of Xuzhou Medical University, but restrictions apply to the availability of these data, which were used under license for the current study, and so are not publicly available. Data are however available from the authors upon reasonable request and with permission of Affiliated Hospital of Xuzhou Medical University. If someone wants to request the data from this study, please contact the first author at 615,898,830@qq.com.

## Abbreviations

FPFA: Femoral prosthesis flexion angle
TKA: Total knee arthroplasty
CT: computed tomography
KSS: Knee society score
ROM: Range of motion
VAS: Visual analogue scale
BMI: Body mass index
PS: Posterior-stabilized
PCO: Posterior condylar offset
TFA: Anatomical tibiofemoral axis
ICC: Intraclass correlation coefficient
LSD: Least significant difference
